# *Ex Vivo* Expanded Human NK Cells Survive and Proliferate in Humanized Mice with Autologous Human Immune Cells

**DOI:** 10.1038/s41598-017-12223-8

**Published:** 2017-09-21

**Authors:** Fatemeh Vahedi, Tina Nham, Sophie M. Poznanski, Marianne V. Chew, Mira M. Shenouda, Dean Lee, Ali A. Ashkar

**Affiliations:** 1 Department of Pathology and Molecular Medicine, McMaster Immunology Research Centre, McMaster University, Hamilton, Ontario, Canada; 2 Cellular Therapy and Cancer Immunology Program, Department of Hematology/Oncology and BMT, Nationwide Children’s Hospital, The Ohio State University Comprehensive Cancer Center, Columbus, United States

## Abstract

Adoptive immune cell therapy is emerging as a promising immunotherapy for cancer. Particularly, the adoptive transfer of NK cells has garnered attention due to their natural cytotoxicity against tumor cells and safety upon adoptive transfer to patients. Although strategies exist to efficiently generate large quantities of expanded NK cells *ex vivo*, it remains unknown whether these expanded NK cells can persist and/or proliferate *in vivo* in the absence of exogenous human cytokines. Here, we have examined the adoptive transfer of *ex vivo* expanded human cord blood-derived NK cells into humanized mice reconstituted with autologous human cord blood immune cells. We report that *ex vivo* expanded NK cells are able to survive and possibly proliferate *in vivo* in humanized mice without exogenous cytokine administration, but not in control mice that lack human immune cells. These findings demonstrate that the presence of autologous human immune cells supports the *in vivo* survival of *ex vivo* expanded human NK cells. These results support the application of *ex vivo* expanded NK cells in cancer immunotherapy and provide a translational humanized mouse model to test the lifespan, safety, and functionality of adoptively transferred cells in the presence of autologous human immune cells prior to clinical use.

## Introduction

Since the advent of the cancer immune surveillance concept, the adoptive transfer of immune cells, particularly T cells and natural killer (NK) cells, has emerged as a targeted method of harnessing the immune system against cancer^1^. NK cells have garnered immense attention as a promising immunotherapeutic agent for treating cancers. NK cells are critical to the body’s first line of defense against cancer due to their natural cytotoxicity against malignant cells^2^. NK cell cytotoxic activity is regulated through a balance of activating and inhibitory receptors that enables fine-tuned control of cytotoxic activity, preventing cytotoxicity against healthy cells, while maintaining effective cytotoxic capacity against tumor cells. Indeed, multiple studies have demonstrated the safety of adoptive NK cell transfer and clinical anti-cancer effects, highlighting the potential for NK cells as an effective cancer immunotherapy^3–7^.

Despite their vast therapeutic potential, a major limitation to the development of NK cell therapies has been the lack of efficient methods to generate adequate numbers of NK cells for clinical efficacy. As a result, much research has focused on generating NK cell expansion protocols. NK cells have been expanded from multiple sources, including peripheral blood and umbilical cord blood (CB)^8–11^. *Ex vivo* NK cell expansion methods have been developed using cytokines in combination with artificial antigen-presenting cells (aAPCs) as feeder cells^8,12–14^. Of these *ex vivo* expansion methods, the use of engineered membrane-bound IL-21 K562 (K562-mb-IL21) feeder cells in combination with IL-2 supplementation has demonstrated the greatest fold expansion of NK cells over 21 days. These NK cells also maintain potent cytotoxicity against tumor targets, rendering this method of expansion promising for clinical application^8^.

With the emergence of adoptive immune cell therapies and the generation of efficient *ex vivo* NK cell expansion protocols, there is a need for a translational pre-clinical model in which to test the survival, function, and safety of adoptively transferred immune cells. While studies have assessed the effects of adoptively transferred NK cells in immunodeficient mice and xenograft models^15–17^, these models have limited translational applicability as they lack a functional immune system. Indeed, it would be more prognostic to test the effects of adoptively transferred cells in the context of a human immune system as this more closely reflects a clinical scenario. In this study, using CB-derived NK cells (CB-NK cells) expanded *ex vivo* with K562-mb-IL-21 and IL-2, we demonstrate for the first time that expanded human NK cells survive and proliferate in an autologous human immune system (humanized) mouse model without the need for *in vivo* IL-2 administration. These results support the use of expanded NK cells as a feasible cancer therapy and provide a novel humanized model within which to test the effects of adoptively transferred cells prior to clinical application.

## Results and Discussion

Although NK cells have proven to be a promising candidate for cancer immunotherapy, a remaining limitation of adoptive NK cell therapy is the poor *in vivo* survival of NK cells. Despite the recent advances in K562-mb-IL-21-based *ex vivo* expansion technologies^10^, little is known about the *in vivo* lifespan of expanded NK cells upon adoptive transfer. While previous groups have tested the *in vivo* efficacy of adoptively transferred NK cells using immunodeficient mice^15–17^, these models have several drawbacks. For instance, in order to maintain cell survival, these models require regular cytokine supplementation in the form of IL-2 or IL-15, which are known to cause severe toxicities in clinical application^18,19^. In addition, the lack of human immune system in these mouse models also prevents the study of potential human immune cell-cell interactions^10,15–17^. With these shortcomings in mind, we have developed a pre-clinical model that examines the *in vivo* lifespan of *ex vivo* expanded NK cells through the adoptive transfer of autologous NK cells into humanized mice.

**Figure 1:**
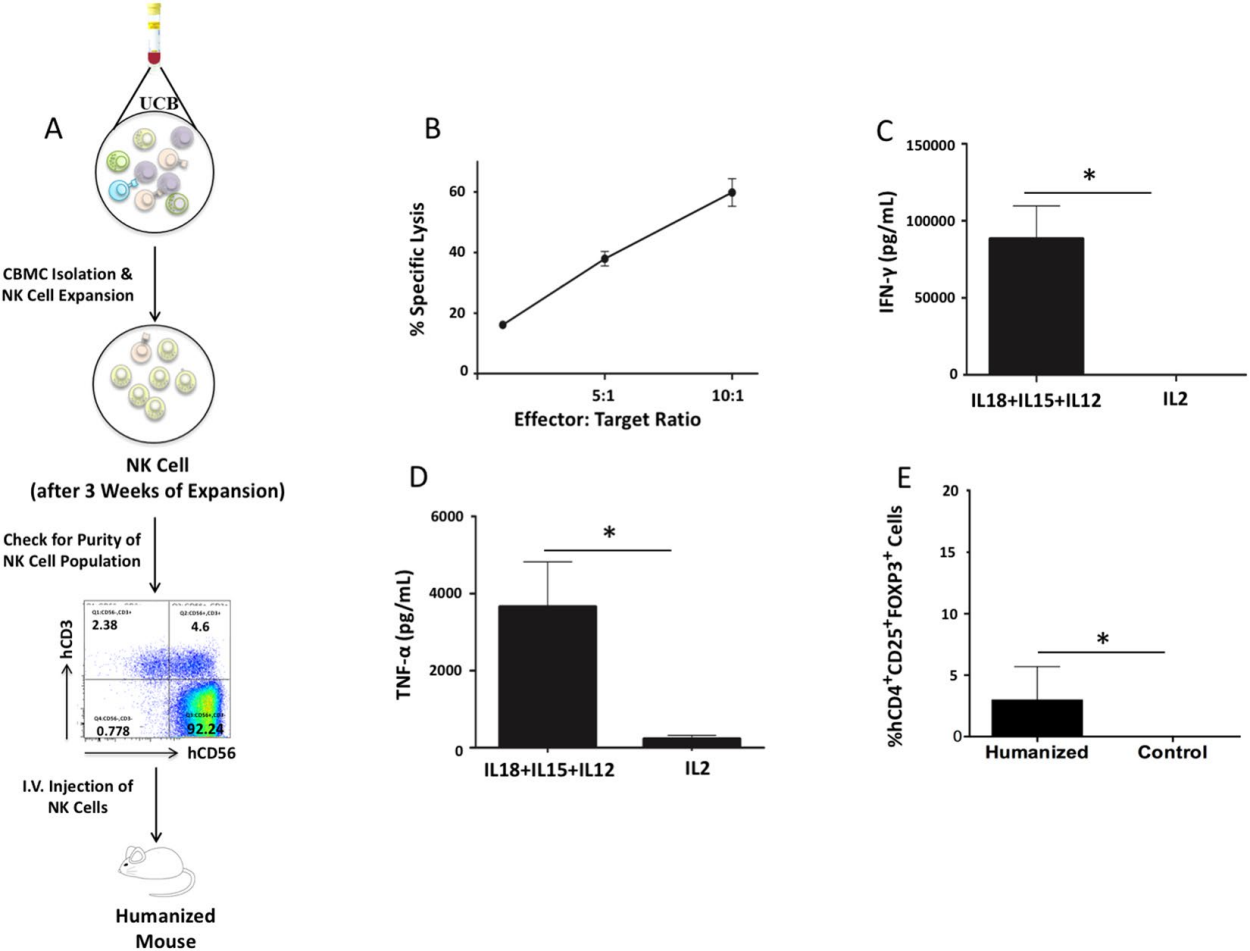
NK cells expanded from CB are cytotoxic and have the capacity to produce pro-inflammatory cytokines. (**A**) One fraction of an autologous CB unit was isolated for cord blood mononuclear cells (CBMCs). CBMCs were subjected to an NK cell expansion process employing K562-mb-IL-21 cells and IL-2. After 3 weeks of expansion, NK cell purity was assessed through flow cytometry. The expanded NK cells were then adoptively transferred into humanized or control NRG mice through i.v. injection. (**B**) Expanded NK cell cytotoxicity was assessed via a cytotoxicity assay against MDA-MB-231-Luciferase target cells (n = 2). Cytotoxicity assay was conducted 2 times. Expanded NK cell (**C**) IFN-γ (n = 3) and (**D**) TNF-α (n = 3) production was assessed via ELISA following stimulation with IL-18 + IL-15 + IL-12 or IL-2. Stimulation and ELISA experiments were repeated for a total of 3 times. Results were analyzed via student’s t-test, *p < 0.05. (**E**) The Treg cell population was evaluated by flow cytometry. The splenocytes of humanized mice (n = 5) were isolated and stained for hCD4, hCD25, and hFOXP3.

We reconstituted NRG mice with a human immune system using CB-derived CD34+ hematopoietic stem cells. Human immune cell reconstitution was established by 12 weeks, with a prominent hCD45+ cell population in the blood (Supplemental Fig. 1). We then further examined hCD45+ immune cell subsets for T cells (hCD3+), B cells (hCD19+) and CD14+ myeloid cells (Table 1). Since the presence of regulatory T cells (Treg) may play an important role in adoptive cell therapy, we have also evaluated the presence of regulatory T cells by staining splenocytes of humanized mice with human regulatory T cells markers (hCD4+ hCD25+ hFOXP3) (Supplemental Fig. 1). We expanded NK cells from a CB unit autologous to that used for immune cell engraftment and once the engraftment of human immune cells in NRG mice was confirmed, expanded NK cells were then adoptively transferred into reconstituted mice and NRG mice without human immune cell reconstitution as controls. The purity of the NK cell culture upon adoptive transfer was >90%, with NK cells defined as CD56+CD3− cells (Fig. 1A). It was important to confirm that the NK cells being adoptively transferred were functional. Thus, we conducted a cytotoxicity assay and assessed their capacity for cytokine production following expansion. Results from the cytotoxicity assay show that the expanded NK cells had cytotoxic activity against MDA-MB-231-Luciferase targets (Fig. 1B). To assess the capacity for cytokine production, NK cells were stimulated with IL-18 + IL-15 + IL-12 which has previously been shown to induce high levels of cytokine production by NK cells^20^. Expanded CB-NK cells produced high levels of IFN-γ (Fig. 1C) and TNF-α (Fig. 1D) following IL-18 + IL-15 + IL-12 stimulation. Thus, adoptively transferred NK cells were cytotoxic and had the capacity to produce high levels of pro-inflammatory cytokines. Further, NK cells expanded using this same expansion system have been found to maintain cytotoxic ability once delivered *in vivo* ^21,22^. Staining of splenocytes of humanized mice showed the presence of Treg cells (hCD4+ hCD25+ hFOXP3) (Fig. 1E).

**Table 1:** Percentage of human immune cell reconstitution in NRG mice 12 weeks post hematopoietic stem cell (HSC) engraftment into liver of newborn pups.

	hCD45	hCD3^a^	hCD4^b^	hCD8^b^	hCD19^a^	hCD14^a^
Blood (n = 5–22)	41.4 ± 12.41	43.75 ± 17.8	60.8 ± 8.2	35.1 ± 5.6	34.4 ± 5.2	10.56 ± 4.23
Spleen (n = 5–7)	52 ± 5.1	32.4 ± 6	40.3 ± 7.2	47.7 ± 4.5	57.9 ± 6.5	0.9 ± 0.2

**Figure 2:**
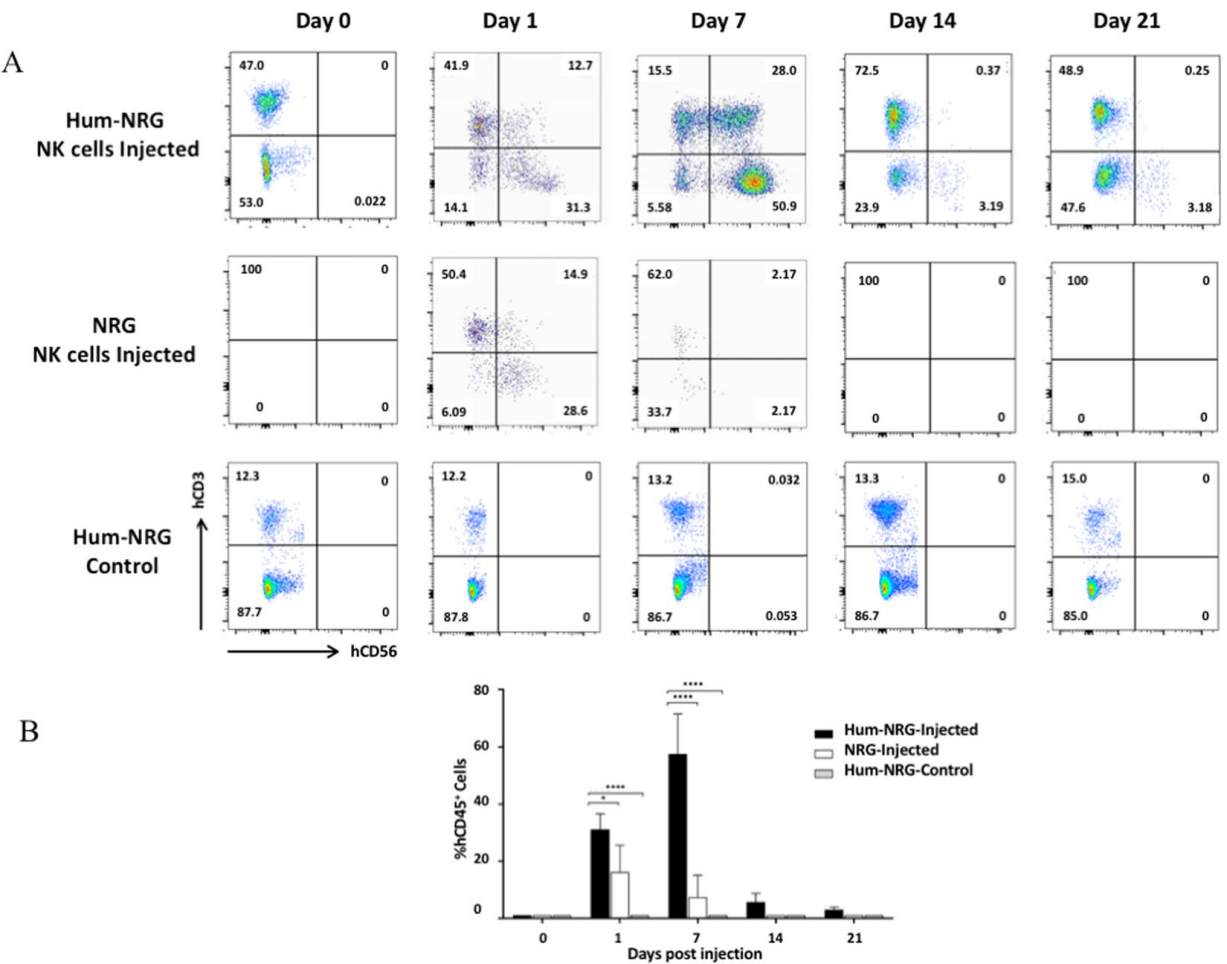
NK cells survive for 21 days after adoptive transfer to autologous humanized mice. *Ex vivo* expanded CB-NK cells were adoptively transferred into either autologous humanized NRG or control mice through i.v. injection. Blood was collected via facial bleeds at the respective time points. PBMCs were stained with anti-mouse hCD45, hCD3 and hCD56. The hCD45+ population was analyzed using a cross gate on hCD3 and hCD56. NK cells were defined as hCD56^+^hCD3^−^ events. (**A**) Representative flow cytometry plots of human lymphocyte populations at days 0, 1, 7, 14 and 21 days post cell transfer. Groups included humanized mice that received an autologous NK cell transfer, NRG mice that received the same NK cells and humanized mice that did not receive any cells as controls for baseline populations. (**B**) The percentage of hCD56+ hCD3− cells in humanized and control NRG mice after adoptive transfer was analyzed. The experiment was conducted twice, using 4 mice per group each time. Results were analyzed via two-way ANOVA with Bonferroni multiple comparisons.

Following adoptive transfer of autologous *ex vivo* expanded NK cells, survival was assessed at indicated days in the blood (Fig. 2A). It is known that humanized mice have minimal levels of NK cell reconstitution upon human immune cell engraftment without IL-15 trans-presentation^22^, which we confirmed in our control humanized mice that did not receive adoptively transferred NK cells (Fig. 2A). Therefore, the presence of CD56^+^CD3^−^ NK cells on Day 1 is solely attributed to the adoptive transfer of expanded NK cells. By day 7 following adoptive NK cell transfer, the expanded NK cell population was almost entirely diminished in control NRG mice, but persisted in humanized mice. In the context of an allogeneic NK cell transfer, it was observed that NK cells showed no evidence of proliferation *in vivo* and did not persist beyond 14 days post-transfer (Supplemental Fig. 2). An unexpected observation in the humanized mice post-NK cell transfer was evidence of NK cell proliferation, since the proportion of hCD56^+^ NK cells almost doubled by day 7 (Fig. 2A and B) and the mean fluorescence intensity of CD56 increased compared to day 1, which also occurs when these cells begin to proliferate *in vitro* (data not shown). Expanded NK cells also remained detectable until at least Day 21 in humanized mice, whereas they were absent in control NRG mice by Day 14 (Fig. 2A and B). Previously, high-dose cytokine administration was required to maintain human NK cell persistence in *in vivo* models^10,15–17^; however, our findings demonstrate that NK cells *ex vivo* expanded using K562-mb-IL-21 cells and IL-2 have the capacity to survive and proliferate without the need for exogenous cytokine administration when autologous human immune cells are present in the humanized mouse model. Therefore, the presence of autologous human peripheral blood mononuclear cells (PBMCs) may support survival and proliferation of expanded NK cells *in vivo*, which negates the need for exogenous cytokine supplementation.

To our knowledge, this is the first study to show that adoptively transferred *ex vivo* expanded NK cells survive and possibly proliferate *in vivo* in the presence of autologous immune cells without exogenous cytokines and presents a novel humanized mouse model for autologous NK cell immunotherapy. Our findings demonstrate that *ex vivo* NK cell expansion with K562-mb-IL-21 cells is promising for adoptive NK cell therapy, as it generates expanded, functional NK cells that can survive and possibly proliferate upon adoptive transfer to an autologous recipient, without any form of cytokine administration. The survival and proliferation of NK cells in this model could be due to the support of autologous human immune cells, particularly through cell-cell interactions and the presence of endogenous human IL-15 and IL-2^22,23^. The ability of NK cells to survive independent of exogenous, high-dose cytokines would be beneficial in clinical applications since these cytokines often have toxic and off-target effects^18,19^. While the presence of supporting human cell types is a strength of this model, it also produces a limitation. In the context of tumor challenge, for example, the presence of human T cells in the humanized mice would make it difficult to delineate the effect of transferred NK cells. Here, T cell depletion would be required in order to characterize the role of NK cells in tumor destruction. Regardless, this humanized mouse model is advantageous for the study of autologous adoptive cell transfer compared to past models for several reasons. First, it does not require repeated, high-dose cytokine supplementation to maintain adoptive NK cell survival. Second, the presence of autologous PBMCs allows for the analysis of human immune cell-cell interactions and more closely represents clinical adoptive cell transfer. In turn, this model may be used to test the safety, functionality, and efficacy of adoptively transferred cells prior to clinical application. Finally, the use of this autologous humanized mouse model can be extended from expanded NK cells to study alternative therapeutic cells for adoptive cell immunotherapy.

## Methods

### Ethics statement

All animal experiments were performed in accordance with the Canadian Council on Animal care (CCAC) guidelines and regulations and under the McMaster University Animal Research Ethics Board (AREB) approval. Human CB samples were collected with informed and written consent. All experiments involving human CB were performed in accordance with Canadian ethic guidelines and regulations on the use of human tissue for research and approved by the Hamilton Integrated Research Ethics Board (HiREB).

### Generation of humanized mice

Human CB was collected with informed consent after delivery (Department of Obstetrics and Gynecology, McMaster Children’s Hospital, Hamilton, ON) and used for immune cell reconstitution to generate humanized mice^24^, as illustrated in Figure S1. Two to three mL of each CB unit were processed to isolate CBMCs and cryopreserved for future autologous NK cell expansion. For the generation of humanized mice, enrichment of human CD34+ hematopoietic stem cells (HSCs) was performed by negative immunodepletion of CD2, CD3, CD14, CD16, CD19, CD24, CD56, CD66b and glycophorin A cells using a commercially available kit (RosetteSep, StemCell Technologies, Vancouver, BC, Canada), followed by Lymphoprep density gradient centrifugation. The isolated cells were cryopreserved in freezing media containing 90% FBS and 10% Dimethyl sulfoxide (DMSO) and then stored in liquid nitrogen. NOD/RAG-1.gc−/−(NRG) mice (The Jackson Laboratory, Bar Harbor, ME) were bred in-house and maintained in a specific pathogen free facility. NRG mouse pups, 1–3 days of age, were irradiated using a gamma irradiator (Gammacell 3000) with two doses of 9 cGy separated by a 3 hour interval. Immediately after the second dose of irradiation, 1 × 10^6^ to 2 × 10^6^ freshly thawed HSC cells or freshly isolated cells were resuspended in 30–40 μl of Phosphate Buffered Saline (PBS) and injected intrahepatically with a 30 G needle. Each CB sample yielded enough stem cells to reconstitute up to 5–7 pups. The pups were then weaned at 21 days of age. Twelve weeks following HSC engraftment, blood was analyzed for human leukocyte reconstitution by assessment of human CD45+ cells. Mice with human CD45 levels greater than 15% were selected for experiments. Human CD3+, hCD56+, and hCD14+ subpopulations were analyzed by flow cytometry to identify T lymphocytes, NK cells, and monocytes, respectively. The presence of Treg cells was also evaluated, but as the small amounts of collected blood could not provide enough cells to evaluate Treg cells, the splenocytes of 5 humanized mice were isolated, stained for hCD4, hCD25, and hFOXP3 and checked by flow cytometry.

### *Ex vivo* NK cell expansion and adoptive transfer

CBMCs were cultured with twice the amount of irradiated K562-mb-IL-21 feeder cells and 100 U/mL IL-2 supplementation for *ex vivo* NK cell expansion, as previously described^10^. The co-cultures were hemi-depleted and replenished with IL-2 every 2–3 days and irradiated K562-mb-IL-21 feeder cells were replenished on a weekly basis at a 2:1 (feeder cell: CBMC) ratio. After 21 days of expansion, NK cells were washed to remove IL-2 and 1 × 10^7^ *ex vivo* expanded human NK cells were adoptively transferred into either autologous humanized mice 12 weeks post-engraftment or control NRG mice via tail vein injection, as illustrated in Fig. 1A. NK cell survival and proliferation was analyzed in the blood at days 1, 7, 14 and 21 post-adoptive transfer by flow cytometry (Fig. 2A). Autologous transfer was performed on two separate occasions using a different CB donor each time (2 donors total).

### NK cell stimulation and cytotoxicity assay

After 21 days of *in vitro* expansion, 2 × 10^5^ CB-NK cells were stimulated for 24 hours with IL-18 (100 ng/mL) (MBL, Japan), IL-15 (20 ng/mL), and IL-12 (10 ng/mL), or IL-2 (100 U/mL) (Peprotech, Burlington, Canada). Supernatants were collected at 24 hours and used for IFN-γ and TNF-α ELISAs (R&D Systems) which were conducted according to the manufacturer’s protocol. A cytotoxicity assay was conducted for IL-2-stimulated NK cells using MDA-MB-231-Luciferase target cells as previously described^25^, except fixable viability dye-eFluor 780 (1:1000 dilution) (eBioscience) was used to determine viability.

### Statistical Analysis

Statistical analysis was performed using Graph Pad Prism software (San Diego, USA). Graphs comparing two conditions were analyzed via unpaired student’s t-test. Graphs comparing more than two conditions were analyzed via one-way ANOVA followed by Bonferroni multiple comparisons.

## Electronic supplementary material

Supplementary Information
