# Supplementary material for: Ex Vivo Expanded Human NK Cells Survive and Proliferate in Humanized Mice with Autologous Human Immune Cells
Source: Sci Rep. 2017 Sep 21;7:12083. doi: 10.1038/s41598-017-12223-8 (PMC5608690; doi:10.1038/s41598-017-12223-8)
Supplement: Supplementary file 1 — Supplementary Information [file 41598_2017_12223_MOESM1_ESM.pdf]

Ex Vivo Expanded Human NK Cells Survive and Proliferate in Humanized Mice with Autologous Human Immune Cells

Fatemeh Vahedi[1], Tina Nham[1], Sophie Poznanski[1], Marianne V. Chew[1], Mira M. Shenouda[1], Dean Lee[2], Ashkar A. Ali[1]*

[1]Department of Pathology and Molecular Medicine, McMaster Immunology Research Centre, McMaster University, Hamilton, Ontario, Canada. [2]Cellular Therapy and Cancer Immunology Program, Department of Hematology/Oncology and BMT, Nationwide Children's Hospital, The Ohio State University Comprehensive Cancer Center

Short title: Expanded NK cell survival in autologous humanized mice

*Corresponding author: Ali A. Ashkar, 1280 Main Street West, MDCL 4015, Email Address: ashkara@mcmaster.ca

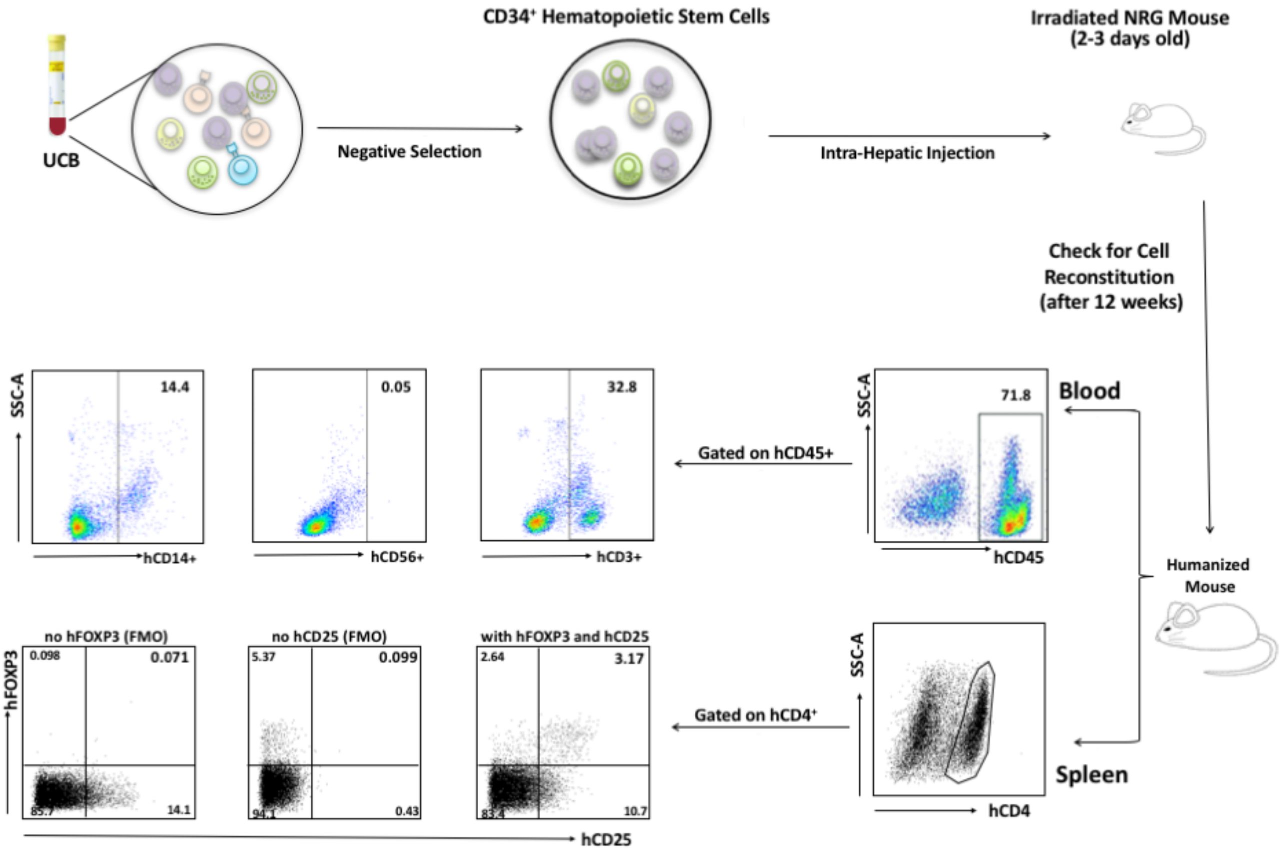

Figure S1. Generation of humanized mice for adoptive transfer of autologous ex vivo expanded human NK cells. Two fractions of an autologous cord blood (CB) unit were either enriched for CD34+ hematopoietic stem cells or isolated for cord blood mononuclear cells (CBMCs). CD34+ hematopoietic stem cells were injected into the liver of irradiated NRG mice at 2-3 days of age. After 12 weeks, facial bleeds were performed and PBMCs were analyzed for hCD45+ cells through flow cytometry to check for the engraftment of human immune cells. Sub-populations of hCD3+, hCD56+, and CD14+ were measured by gating first on the hCD45+ population. The splenocytes of humanized mice(n=5) were isolated and stained for hCD4, hCD25, and hFOXP3 markers to check the Treg cell population. The percentage of CD4+CD25+FOXP3+ population was found to be 2.974 ± 1.214, significantly (student's t-test, $p<0.01$) higher than control mice (NRG, non-humanized). Representative flow cytometry plots of the CD4+CD25+FOXP3+ population are shown.

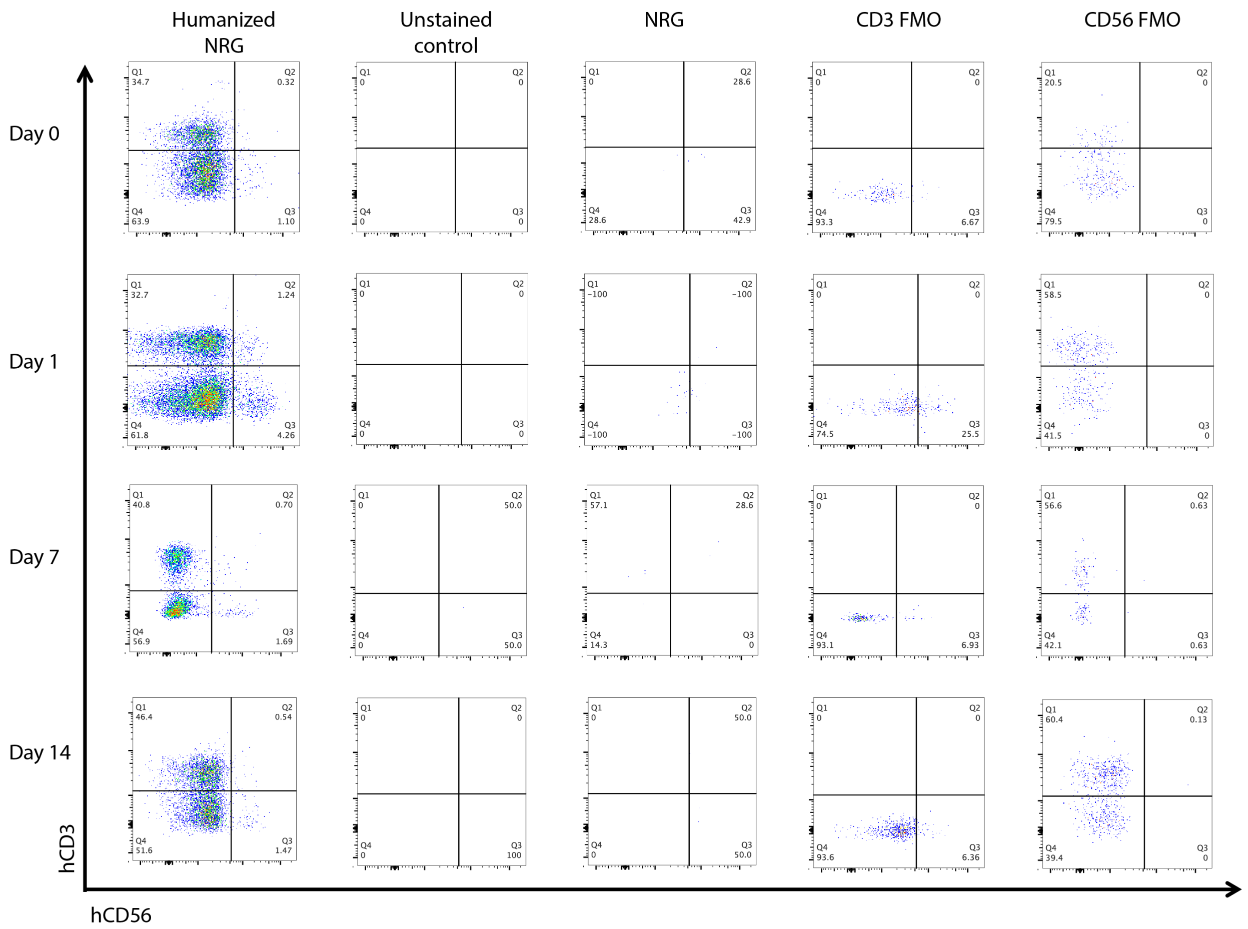

Figure S2. NK cells do not persist following adoptive transfer to allogenic humanized mice. While an increased percentage of NK cells was detected at one day post injection, there was no evidence of NK cells proliferation by day 7 as observed in the autologous transfer and cells were not detectable beyond day 14.
